# Perspective: National Football League Teams Need Chief Diversity Officers

**DOI:** 10.3389/fspor.2022.891516

**Published:** 2022-05-12

**Authors:** Anne L. DeMartini, Barbara Nalani Butler

**Affiliations:** Department of Exercise Science and Sport Management, Kennesaw State University, Kennesaw, GA, United States

**Keywords:** diversity and inclusion, National Football League (NFL), Chief Diversity Officers, sport management, professional sport, diversity equity and inclusion (DEI)

## Abstract

The National Football League (NFL) and its teams, some of the world's most profitable sporting properties, face challenges with diversity, equity, and inclusion (DEI). With a history of discriminatory work environments, including a recent high-profile lawsuit, the NFL and its teams have a poor reputation on these issues. This perspective piece investigated NFL teams' utilization of organization employees dedicated to DEI. Utilizing a content analysis of publicly available data, this piece investigated DEI employees at NFL team organizations. The study analyzed the position's characteristics including the name of the role, the department in which it was housed, and the reporting structure. The study also examined the demographics and professional background of the employees in the roles. The findings conclude that NFL teams lag behind other American businesses in their adoption of Chief Diversity Officer (CDO) roles. As of 2022, only 31.25% of NFL teams had a dedicated DEI staff person. Three additional teams host diversity councils utilizing employees with other job responsibilities. The employees filling the CDO roles were majority women and majority Black. Though not the only answer to a complex problem, in order to address these challenges and move forward, the NFL teams should create Chief Diversity Officer (CDO) roles. These positions should have appropriate reporting relationships, well-crafted position responsibilities, generous resources, and qualified and experienced employees.

## Introduction

### National Football League

The National Football League (NFL) is the most powerful organization in American sport (Crepeau, 2020) and is also “one of the most significant engines of contemporary culture” (Oates et al., 2014) (p. 3). The NFL, one of the most popular and profitable sporting leagues, dominates American sports' viewership ratings (Oates et al., 2014; McGannon and Butryn, 2020). The popularity of American football, along with the massive influx of media dollars, has driven the value of individual NFL franchises to billions of dollars (Crepeau, 2020).

The NFL and its teams have struggled with issues of diversity, equity and inclusion (DEI) in the past including the use of racist mascots (Bruyneel, 2016), race norming (Hobson, 2021), and perpetuating discriminatory work environments (Eichhorn, 2020; Shephard, 2021). The NFL generates billions of dollars, to the benefit of its team owners, whose lack of diversity is striking, given the racial makeup of the players in the league (McGannon and Butryn, 2020). There was not a non-white NFL team owner until 2012, and there are currently only two owners of color with major ownership interests and significant involvement in the operations of an NFL team (Lapchick, 2020). Team CEO/President positions have been predominantly held by white men (Lapchick, 2020).

Commentators have criticized the NFL's attempts to address DEI and social justice issues. Researchers analyzed the NFL and its teams' responses to athlete activism. Montez de Oca characterized the league's reaction to Black players' activism as contradictory attempts to appease fans and its mostly Black on-field workers (Montez de Oca, 2021). Rugg argued that the league tried to control the voice of rebellious Black players by subsuming their social justice efforts into a weak, market-friendly version of “justice” based in calls for unity (Rugg, 2019). Niven found that NFL teams punished athletes who protested during games, paying them less and treating them worse than their peers (Niven, 2020).

Most of the literature focuses on NFL coaches and players rather than administrative executives. Scanga identified the lack of diversity among decision makers, the absence of diversity in the final candidate choices, and the anti-tampering policy as factors facilitating and perpetuating a cycle of racial employment discrimination in the National Football League (Scanga, 2004). Conlin and Emerson discovered strong evidence that non-white players face hiring discrimination in the NFL, though they are treated more equitably in retention and promotion decisions (Conlin and Emerson, 2006). Madden found Black coaching candidates were held to a higher standard during the hiring process (Madden, 2004). Some investigators have found that the institution of the Rooney rule may have mitigated that discrimination (Fanning Madden and Ruther, 2010; DuBois, 2016). The Rooney rule requires NFL teams to interview at least one minority candidate for any head coaching vacancy (DuBois, 2016). However, Pitts et al. recently determined after accounting for numerous characteristics of coordinators, Black coordinators were significantly less likely than non-Black coordinators to become head coaches over the 2018–2020 seasons (Pitts et al., 2022).

Very recently, the public eye again focused on the NFL's treatment of DEI issues. Brian Flores, former Miami Dolphins head coach, filed a lawsuit in February 2022 alleging that “the NFL remains rife with racism, particularly when it comes to the hiring and retention of Black Head Coaches, Coordinators and General Managers” (para 3) (Smith, 2022). The high-profile lawsuit claims that Flores was subjected to “sham” interviews for head coach positions (Smith, 2022).

Also, a now-former head coach's disparaging emails came to light—containing racist, misogynistic, homophobic, and transphobic content (Razack and McKenzie, 2021). These emails were uncovered by an independent investigation into the Washington Football Team's well-documented misogynistic and toxic culture (Shephard, 2021). Commentators noted the retrograde opinions expressed in the emails, and their wide acceptance among those with whom he shared them, illuminated the extent to which the NFL's efforts to broaden its audience are a sham (Shephard, 2021). Shephard opined these emails as evidence of “deep cultural rot at the center of the NFL and just how far the league still has to go to fix it” (Shephard, 2021) (para 4) and that the NFL “has no sincere interest in the changes its marketing campaign pretends to take seriously” (Shephard, 2021) (para 7). The NFL's “systemic and systematic protection of white men in power has bred hypocrisy, race norming, gender exclusion and violence and performative acts of solidarity with the league's majority racialized player pool” (Razack and McKenzie, 2021) (para 5).

Following a summer of mass political mobilization triggered by the police murder of George Floyd, racial justice activism compelled the NFL to respond to public pressure (Montez de Oca, 2021). In 2020, as an attempt to improve DEI efforts, the NFL made enhancements to the existing Rooney Rule and extended its application from solely coaching and football operations jobs to a wide range of executive positions (Lapchick, 2020). Teams must now include people of color and/or female applicants in the interview processes for senior level front office positions such as club president and senior executives in communications, finance, human resources, legal, football operations, sales, marketing, sponsorship, information technology, and security positions. Additionally, teams that develop women or people of color to be candidates for primary football executive, general manager positions or a head coach position will earn draft pick rewards (Lapchick, 2020). In March 2022, the league announced two enhancements to the Rooney Rule (Jones, 2022). Interviewing a woman for coaching and front office vacancies would count toward fulfilling the Rooney Rule requirement and only interviews conducted in person will count (Jones, 2022).

In 2020, the NFL League Office also hired Jonathan Beane as Senior Vice President and Chief Diversity and Inclusion Officer, highlighting the increased emphasis on continuing the League's progress when it comes to improving diversity and inclusion in its workplace and in all aspects of its business (Lapchick, 2020). The League earned an A+ rating from the 2020 Race and Gender report card for its diversity and inclusion initiatives (Lapchick, 2020).

However, even with the increased efforts at the league level, individual teams continue to struggle with DEI. Richard Lapchick, Director of The Institute for Diversity and Ethics in Sport, noted the continued disparity in racial and gender hiring practices between the NFL's league office and their 32 teams. He identified a serious underrepresentation of women and people of color at the team level in positions with significant decision-making power (Lapchick, 2020). At the team level, only 15.9% of the vice presidents are people of color and only 25.1% are filled by a woman (Lapchick, 2021b). At the beginning of 2021, only 17.3% of C-suite positions at NFL teams were filled by people of color and 28.6% by women (Lapchick, 2021b). By March 2022, all 32 teams are required to fully implement a DEI plan (Lapchick, 2020). One component of those plans should be dedicated DEI staff positions.

### Chief Diversity Officer Roles

Diversity leads to greater financial performance and drives productivity, innovation, and decision making (U.S. Government Publishing Office, 2019). The strategic diversity leadership movement has moved from a narrow focus on access and equity issues to a broader frame that addresses the rich experience of an increasingly diverse, global world. This shift requires organizations to make DEI central to their operation rather than leaving it on the margins (Williams and Wade-Golden, 2013). One way to accomplish that is creating staff roles dedicated to DEI.

Government entities, for-profit and non-profit corporations, and educational institutions have all created the positions, often called Chief Diversity Officers (CDOs). Local governments have been addressing equity issues by adding CDOs or staff with similar titles or focus (Kimbrough, 2017). In recent years, CDOs have assumed an increasingly vital role in higher education. Between 2000 and 2010, at least 60 of the nation's leading College and Universities reframed their senior diversity administrative role (Williams and Wade-Golden, 2013).

There has been significant progress in the creation of these roles in American business. In 2012, only 198 out of 957 (20.7%) Standard and Poor's (S&P) 500 firms had adopted CDO positions (Shi et al., 2018). In that same year, Forbes reported about 60% of Fortune 500 companies had CDOs or executive roles designated for diversity (Kwoh, 2012). As of 2021, about 53% of S&P 500 firms have a CDO position or equivalent, up from 47% in 2018 (Green et al., 2021). Institutional pressures, crucial stakeholders that control important resources, or internal powerful actors may motivate firms to adopt CDO positions (Shi et al., 2018).

Recently, US corporations have been setting hiring records for CDOs and large companies have poached peers for management talent in the DEI space (Green, 2021). After the mass protests in 2020, new hires of CDOs in the S&P 500 index tripled the rate of the previous 16 months, increasing to approximately a dozen each month (Green, 2021). At least 60 other publicly traded firms appointed their very first diversity leader in that time frame (Green, 2021). Eighty-five of the 100 largest American corporations have a CDO, 16 of which added or elevated the role in 2020 or 2021 (Green et al., 2021). The increase in CDOs in large American corporations demonstrates firms' commitment to workforce diversity and attests to their willingness to invest resources to accomplish it (Shi et al., 2018).

However, even with the uptick in hires and recognition of the role's importance, turnover in the role has been high. The average tenure is 3.2 years, compared with 5.5 years for a CEO (Green, 2021). Additionally, since the adoption of the role is relatively new, though it is becoming more common, there is not a long track record of success (Gabriel, 2019).

Definitions of the CDO role vary. These positions lead diversity, inclusion, and equity efforts on several fronts, internally and externally (Kimbrough, 2017). CDOs generally have hybrid job descriptions that include recruitment, human resources, marketing, ethics and legal compliance (Kwoh, 2012). CDO's primary duty is to conceptualize, define, assess, nurture and cultivate diversity (Williams and Wade-Golden, 2007; Shi et al., 2018). The CDO also brings a new perspective to the top management team and can coordinate diversity initiatives and foster relationships with important stakeholders (Shi et al., 2018).

In a higher education context, Williams and Wade-Golden's foundational work defined a CDO as “boundary-spanning senior administrative role that prioritizes diversity-themed organizational change as a shared priority at the highest levels of leadership and governance” (Williams and Wade-Golden, 2013) (Chapter 1, para. 8). Williams and Wade-Golden recommended that as the institution's highest ranking diversity administration they report to the president (Williams and Wade-Golden, 2013). The CDO's goal is to create an environment that is inclusive and excellent for all, which requires them to serve “an integrative role that coordinates, leads, enhances, and…supervises formal diversity capabilities of the institution” (Williams and Wade-Golden, 2013) (Chapter 1, para. 8).

CDO roles place high demands on the employees who hold them. To be effective leaders, CDOs must remain highly involved with their communities and in critical conversations, engaged on the subject matter, not defensive, and be able to hear critiques (Wood, 2021). CDOs must have a broad span of knowledge that can be leveraged in different domains, including a detailed and sensitive understanding of diversity topics across identity groups, ranging from race and ethnicity to sexuality, national origins, disability, and veteran status, among others. CDOs must also retain working knowledge of key affirmative action and federal and state policies (Williams and Wade-Golden, 2013). The success of the CDO depends on personal characteristics of this employee including leadership traits, ability to build networks, and understanding organizational change (Leon, 2014).

In 2012, CDOs holding these roles in Fortune 500 companies were 65% female and 37% African-American (Kwoh, 2012). Their professional backgrounds ranged from human resources and marketing to finance and operations (Kwoh, 2012; Shi et al., 2018). Approximately 25% reported directly to the CEO, while the rest answered to human resources or another department (Kwoh, 2012).

Organizational design also impacts the success of the CDO. Rank, support staff, reporting structure, and resources can support or jeopardize their work (Leon, 2014). Historically, workplace diversity has been the domain of human resource departments and managers responsible for DEI issues were considered to have few career options and negative connotations (Anand and Winters, 2008; Shi et al., 2018). In the past, CDOs were one person, with little to no staff, resources, or direct authority (Hancock, 2018).

To set up a CDO for success, firms must make more substantial resources commitments than for the adoption of other types of diversity programs (Shi et al., 2018). Adding a CDO position to the firm's senior management team represents a significant structural change (Shi et al., 2018). Shi et al. (2018) found most CDOs either belong to the top management team, reporting to the CEO, or report to a top management team member. Creating a new senior position requires investment of people, money, and setting up priorities, goals, channels of communication, and organizational routines that align with the rest of the organization (Shi et al., 2018).

## Methods

This study utilized content analysis of publicly available secondary data. Content analysis defines the process of summarizing and reporting written data (Cohen et al., 2018). Content analysis has become a popular method for qualitative and quantitative analyses in management and international business research (Gaur and Kumar, 2018). Prior studies within the sport management field have also utilized this method (Pederson and Pitts, 2001; Peetz and Reams, 2011; Miller et al., 2018; Williams et al., 2021).

The investigator used Google and the LinkedIn website and entered search terms “[team franchise name]” and diversity, “[team franchise name]” and equity, “[team franchise name]” and inclusion. Investigator also used the Find function for each search term on each NFL team's website's front office staff page. See Table 1 for numbers of search results.

**Table 1:** Number of search results.

	**Google [team franchise**			**LinkedIn [team franchise**			**NFL team website front office**
	**name] and**			**name] and**			**staff directory word search**
	**Diversity**	**Equity**	**Inclusion**	**Diversity**	**Equity**	**Inclusion**	**Diversity, equity or inclusion**
Arizona Cardinals	1,080,000	830,000	479,000	12	11	5	0
Atlanta Falcons	557,000	863,000	460,000	34	27	24	0
Baltimore Ravens	693,000	424,000	634,000	21	14	14	0
Buffalo Bills	633,000	466,000	1,120,000	18	24	16	0
Carolina Panthers	542,000	276,000	439,000	33	24	16	0
Chicago Bears	1,710,000	570,000	717,000	31	34	21	1
Cincinnati Bengals	1,270,000	286,000	1,050,000	16	15	11	0
Cleveland Browns	644,000	383,000	563,000	26	24	18	0
Dallas Cowboys	991,000	608,000	1,060,000	32	35	24	0
Denver Broncos	754,000	511,000	661,000	27	22	15	2
Detroit Lions	401,000	393,000	483,000	29	18	20	1
Green Bay Packers	552,000	545,000	848,000	25	17	17	0
Houston Texans	513,000	335,000	869,000	18	19	13	0
Indianapolis Colts	489,000	277,000	416,000	22	13	22	1
Jacksonville Jaguars	918,000	256,000	747,000	23	21	16	1
Kansas City Chiefs	534,000	473,000	833,000	29	17	17	0
Las Vegas Raiders	383,000	323,000	366,000	9	4	6	0
Los Angeles Chargers	199,000	492,000	729,000	11	6	7	1
Los Angeles Rams	630,000	350,000	475,000	17	87	12	0
Miami Dolphins	669,000	446,000	592,000	39	39	31	0
Minnesota Vikings	1,180,000	1,020,000	518,000	24	19	15	1
New England Patriots	1,130,000	744,000	947,000	11	39	9	0
New Orleans Saints	976,000	316,000	593,000	13	26	7	0
New York Giants	1,410,000	591,000	1,380,000	6	11	2	0
New York Jets	1,240,000	1,020,000	703,000	21	64	23	0
Philadelphia Eagles	741,000	1,010,000	681,000	96	42	46	1
Pittsburgh Steelers	694,000	1,020,000	1,460,000	10	21	7	0
San Francisco 49ers	727,000	11,090,000	640,000	30	48	22	1
Seattle Seahawks	605,000	447,000	597,000	38	41	20	1
Tampa Bay Buccaneers	437,000	375,000	542,000	28	15	13	0
Tennessee Titans	410,000	665,000	360,000	13	7	9	0
Washington Football Team	356,000	362,000	325,000	22	12	11	
Washington Commanders	151,000	66,700	139,000	8	6	7	0

After identification of the existence of a DEI role on the NFL team staff and the name of the person holding that position, the investigator then confirmed the role by using the person's name as a search term on LinkedIn and google. Utilizing the LinkedIn profile and any biographical information found through Google, the investigator coded the available information. The measures used for coding were position (job title, department, reporting structure), education (highest degree earned, degree discipline) and prior professional background and qualifications.

The investigator also coded the photos for demographic characteristics. Visual images can be analyzed similarly to analyzing texts, for example, through “reading” the meanings (Cohen et al., 2018). Since these characteristics were not self-reported, the investigator was required to perceive them.

The study utilized the ethnicity (Hispanic/Latino/a/x/e or not Hispanic/Latino/a/x/e) and race (White, Black. American Indian, Asian or Native Hawaiian/Pacific Islander) categories as based on the US Office of Management and Budget's Revisions to the Standards for the Classification of Federal Data on Race and Ethnicity (Department of Health Human Services Office of Minority Health, 2018). The investigator observed socially assigned race, the racial/ethnic categorization of individuals by others (White et al., 2020). Appearance-based observed race is an external classification based solely on readily observable characteristics (Roth, 2016). This includes not only a person's phenotype but also visible status markers, clothing, hairstyle, and the context of the observation (Roth, 2016). These classifications largely reflect how perceptions by the dominant or mainstream social groups (White et al., 2020). In social research, it is typically measured by the interviewer's classification of the individual (Roth, 2016).

The investigator determined gender using phenotype and dress. “Determining gender” is the umbrella term for social practices of placing others in gender categories (Westbrook and Schilt, 2013). People present information about their gender and others then interpret this information, placing them in gender categories (Westbrook and Schilt, 2013). The process of gender determination often relies on visual and behavioral cues (Westbrook and Schilt, 2013) that relate to society's expectations of masculine vs. feminine behavior and presentation (Caffrey, 2021).

## Findings

Out of the 32 NFL franchises, 10 (31.25%) had identifiable DEI dedicated staff roles like a CDO. See Table 2. The Jacksonville Jaguars, Chicago Bears, Detroit Lions and Seattle SeaHawks had more than one executive DEI dedicated position. There was evidence three additional teams, the Los Angeles Rams, the New York Jets, and the Miami Dolphins, have created DEI councils utilizing employees who maintained other primary roles.

**Table 2:** NFL teams with CDO.

**Team name**	**Title of position**	**Reports to**	**Highest degree**	**Discipline**	**Race**	**Gender**	**Background**
Arizona Cardinals	Chief People Officer	owner	MBA	Human Resources Leadership	Black	Male	Corporate
Chicago Bears^*^	SVP diversity, equity and inclusion	President/CEO	Bachelors	Business	Black	Female	Intra-organization events and entertainment; intercollegiate athletics administration
Denver Broncos	VP of diversity, equity and inclusion	SVP of strategy	EdD, masters	Athletic administration	Black	Female	Coaching, intercollegiate athletics administration
Detroit Lions^*^	SVP chief people officer and diversity officer	President/CEO	BFA	Musical theater	White	Female	Corporate; technology
Indianapolis Colts	Director of diversity, equity and inclusion	VP of finance	MS, EdD	Kinesiology; higher education and student affairs	Black	Male	Higher education
Jacksonville Jaguars^*^	SVP and Chief Community Impact Officer	President/CEO	MS	Business administration	Black	Female	Higher education
Los Angeles Chargers	Director of Latino/a diversity and cultural affairs	SVP, communications and external affairs	Bachelors	Liberal studies	Latina	Female	State government
Minnesota Vikings	Director of inclusion and employee investment	EVP and chief people and culture officer	Bachelors	French	White	Female	Intra-organization since 2006
San Francisco 49ers	Director of diversity, equity and inclusion	VP Human Resources	MSHRM	Human resource management	Black	Female	Corporate
Seattle Seahawks^*^	VP of diversity, equity and inclusion	President/CEO	Bachelors	Communications	Black	Female	Corporate

* *Indicates evidence of more than one DEI executive*.

The most common position title was Director/Vice President of Diversity, Equity and Inclusion, with half of teams using this language. Other position titles included Chief People Officer, Vice President of Social of Responsibility and Impact, Director of Latino/a Diversity and Cultural Affairs, and Director of Inclusion and Employee Investment. For positions that were not C-suite level, the most common department in which these positions were housed was Human Resources (also called People and Culture). Other departments represented included Marketing, Strategy, and Communications.

Seven of the employees holding these DEI positions were Black (70%), 20% white and 10% Latina. Eight of the employees were female (80%), 20% male. Five of the ten the employees in these positions held degrees more advanced than Bachelor's degrees. Twenty (20%) percent of the group attained Ed.D degrees and there were examples of an MBA, an MSHRM, and an MS in Sports Administration. Four of the 10 (40%) of the employees held DEI specific training credentials, the most common of which is the University of South Florida's Corporate Training and Professional Certification Diversity, Equity and Inclusion in the Workplace.

The employees in these roles illustrate two dominant pathways to CDO positions. Four of the 10 had previous professional backgrounds in corporate, for-profit businesses including WalMart, Sephora, and Alaska Airlines. The other prominent path was gaining experience in higher education and intercollegiate athletics administration. Most were external hires, with only two of the 10 having spent significant time with the NFL team before assuming the CDO role.

## Discussion

### Results

The finding that only 31.25% of NFL franchises have a CDO demonstrates that NFL teams are significantly behind other American businesses. Even in 2012, well before the protests following George Floyd's murder that prompted so much corporate action, about 60% of Fortune 500 companies had CDOs or equivalent (Kwoh, 2012). Almost a decade later, the NFL's teams have still not caught up to that level. Including diversity councils, the number increases to 40.6%. The executive diversity committee may be less ideal for generating diversity strategy because of its potential lack of expertise and the non-representative nature of the group (Williams, 2013).

That many of the CDO positions were housed in human resources is consistent with previous findings. Historically, human resource departments have supervised workforce diversity (Shi et al., 2018). Similarly, 75% of CDOs at Fortune 500 companies in 2012 answered to human resources or another department (Kwoh, 2012).

Half of the NFL teams' CDOs did not report directly to the owner, president, or CEO. Having a direct line to the CEO can give a CDO more power and visibility (Kwoh, 2012). Most CDOs either report to the CEO or report to a top management team member (Shi et al., 2018). This indicates that NFL teams may not be establishing an organizational structure that will best support the CDOs work. A reporting structure that connects the CDO to the president sends a powerful message to the entire organization and allows CDOs to raise issues within the highest levels of leadership (Williams and Wade-Golden, 2013).

The demographics of the employees who hold these positions did show some progress. In 2012, 65% of Fortune 500 CDOs were female, and 37% were Black (Kwoh, 2012). Most organizations choose CDOs from underrepresented minority groups (Ng et al., 2021). The NFL team's CDOs are 80% female and significantly more racially diverse than the Fortune 500 CDOs, with 70% Black employees. The employees in these positions are significantly more diverse than other executive roles within NFL teams. Only 17.3% of C-suite positions at NFL teams are filled by people of color and 28.6% by women (Lapchick, 2021b). Team senior administration, which includes director and senior manager roles, are 20.1% people of color and 25.3% female (Lapchick, 2021b). Employees in CDO roles do align with the NFL team's production labor racial demographics, as 70.7% of NFL players are Black (Lapchick, 2021b).

The educational and professional background of the NFL team's CDOs indicate that they do take the employee's qualifications seriously. Fifty (50%) percent of the employees had advanced degrees and four of the 10 had DEI specific certifications or credentials. This is different than in the past when some organizations placed employees into the diversity role who had no previous experience, or whose careers were in decline, or someone who happened to be a visible minority with a passion for diversity (Dexter, 2010).

The NFL team's reticence in hiring CDOs may be explained by the NFL's culture and insularity. It is a league run, owned, and coached by a handful of executives which enables systemic oppression (Razack and McKenzie, 2021). Shi et al. (2018) found a strong effect of female top management team representation on firms' likelihood of adopting CDOs. At the beginning of the 2021 season there were only four women in a CEO/President position of an NFL team (Lapchick, 2021b). The number of women in CEO/President positions has increased from zero in 2017 to one in 2018 and 2019 to two in 2020 and four in 2021 (Lapchick, 2021b). At the team level, only 25.1% of Vice President positions are filled by a woman (Lapchick, 2021b). Therefore, the lack of gender diversity in top management teams may lead to a lack of will to develop and support CDOs.

### Recommendations

Organizations may adopt a CDO position if doing so would allow them to increase legitimacy, improve efficiency, change the corporate culture, and increase control over external resources and external actors, in response to diversity-specific pressures (Nath and Mahajan, 2008; Menz, 2012; Shi et al., 2018). The NFL is experiencing diversity-specific pressures and calls to prove that their expressed commitment to diversity and social justice is more than just lip service. Also, Shi et al. (2018) found accumulative industry adoptions impacted firms' decision to adopt CDOs. The NFL teams may feel pressure from other major American professional sports teams, like NBA teams, that are perceived as doing better in terms of diverse hiring and social justice initiatives (Lapchick, 2020, 2021a; Beard, 2021; Butler et al., 2022). Therefore, NFL teams that do not have staff dedicated to DEI should create CDO positions.

CDOs advance an organization's diversity agenda but cannot be solely responsible for transforming an organization's culture (Gabriel, 2019). If firms adopt CDOs simply for impression management, it can waste resources (Shi et al., 2018). Creating a new leadership position sends a strong signal, but it takes more than one executive to make an impact in the face of institutional pushback (Green, 2021). Therefore, these roles deserve support from the owner and CEO/President, appropriate reporting relationships, well-crafted position responsibilities, generous resources, and talented employees.

NFL teams with current CDO positions should audit that role's reporting structure. All NFL team's CDOs should report to the President/CEO, even though the trend of developing new roles at the senior level is fairly recent (Williams and Wade-Golden, 2013). Allowing the CDO to report directly to the President/CEO gives credibility, indicates the value the organization places on the role, and places final accountability with the President/CEO. Businesses characterized as DEI high performers were twice as likely as low performers to report that the CEO/President is primarily responsible for DEI issues (HR Research Institute, 2021). Reporting to the President/CEO, allows the CDO to strengthen alliances and networks across different levels of the organization's hierarchy (Leon, 2014). Raising the profile of the CDO reflects an organization's willingness to expose and close long-standing equity gaps (Zalaznick, 2020).

To truly shift the organization's structure and strengthen its commitment to DEI, the CDO needs to supervise other DEI staff across divisions. The development of a vertically integrated CDO division offers a powerful way of creating a more responsive organizational infrastructure by creating economic, organizational, and strategic effects (Williams and Wade-Golden, 2013). Diversity management has evolved out from under the traditional human resources and talent acquisition roles, to assume more dotted-line responsibilities including corporate strategy, corporate social responsibility, organizational design and effectiveness, corporate marketing and even sales (LLopis, 2011). Change rarely happens when a diversity leader is separated from other departments (Green, 2021).

Since many of the NFL teams have already created diversity councils, teams could ensure that each division is represented on these councils, designate that person as the “Diversity lead” and allocate some percentage of their workload to DEI work. Then, each of these leads report to the CDO as well as their division head. Relying on diversity committees without a CDO can be ineffective, since they are commonly not supported by senior leadership or a true institutional commitment to producing results (Williams, 2013).

The CDOs job description should be carefully drafted. The position's designers should delineate an area of work they define as “strategic diversity leadership work” (Williams and Wade-Golden, 2013). Changing culture and making progress on DEI issues is not easy nor fast (Hancock, 2018). Achieving these goals requires expanding expectations for CDOs outside of tactical areas like compliance, training, problem solving, recruiting, and event planning (Hancock, 2018). CDOs must learn to play a more integral strategic role in the design of new business models, including operating more holistically in a general management and operational capacity (LLopis, 2011). Effective CDOs work to ensure that a DEI lens is rooted throughout all functional areas (internal) and the supply chain (external) (LLopis, 2011).

Once installed, diversity chiefs often face challenges such as lack of budget and direct reports (Green, 2021). NFL teams should ensure CDOs receive the support they need. Organizations must be prepared to give CDOs the resources necessary to succeed (Geisler, 2021). CDOs should be part of strategic planning and all employees should know that the CDO is a power player and not just the organization's conscience (Geisler, 2021). Fully empowering the CDO allows them to leverage diversity as an integral part of the organization's overall strategy (Shi et al., 2018).

Finding the right hire for a CDO position is difficult, because it requires a wide range of competencies (Williams and Wade-Golden, 2013). NFL teams should vet candidates carefully and focus the search on those who can lead and guide change. If not, the teams risk hiring employees who are underprepared for the demands of such a complex, high-profile, and politically charged position (Williams and Wade-Golden, 2013). When an area of responsibility becomes critically important, organizations specialists who are content experts (Williams and Wade-Golden, 2013). The NFL should continue the practice of external hires who have a professional background in corporate DEI.

“The development of the CDO role marks a step toward creating specialized capacity to engage in strategic diversity leadership work” (Williams and Wade-Golden, 2013) (Chapter 1, para. 70). Adopting CDO positions leads stakeholders to perceive organizations are strongly committed to workforce diversity and can be considered role models (Shi et al., 2018). It is time for NFL teams to catch up with American businesses and truly prioritize diversity, equity and inclusion. Though not the only answer to a complex problem, NFL teams should add or modify existing CDO roles to ensure an organization-wide commitment.

## Data Availability Statement

The original contributions presented in the study are included in the article/supplementary material, further inquiries can be directed to the corresponding author.

## Author Contributions

AD and BB contributed to conception and design of the study. AD conducted the content analysis and wrote the first draft of the manuscript. All authors contributed to manuscript revision, read, and approved the submitted version.

## Funding

The publication fee was funded by WellStar College of Health and Human Services at Kennesaw State University for this work.

## Conflict of Interest

The authors declare that the research was conducted in the absence of any commercial or financial relationships that could be construed as a potential conflict of interest.

## Publisher's Note

All claims expressed in this article are solely those of the authors and do not necessarily represent those of their affiliated organizations, or those of the publisher, the editors and the reviewers. Any product that may be evaluated in this article, or claim that may be made by its manufacturer, is not guaranteed or endorsed by the publisher.
